# Cannabis Use during Pregnancy: An Update

**DOI:** 10.3390/medicina60101691

**Published:** 2024-10-15

**Authors:** Angeliki Gerede, Sofoklis Stavros, Christos Chatzakis, Eleftherios Vavoulidis, Panagiota Papasozomenou, Ekaterini Domali, Konstantinos Nikolettos, Efthymios Oikonomou, Anastasios Potiris, Panagiotis Tsikouras, Nikolaos Nikolettos

**Affiliations:** 1 Unit of Maternal-Fetal-Medicine, Department of Obstetrics and Gynecology, Medical School, Democritus University of Thrake, 8100 Alexandroupolis, Greece; k.nikolettos@yahoo.gr (K.N.); eftoikonomou@outlook.com (E.O.); ptsikour@med.duth.gr (P.T.); nnikolet@med.duth.gr (N.N.); 2 Third Department of Obstetrics and Gynecology, Medical School, National and Kapodistrian University of Athens, 11527 Athens, Greece; sfstavrou@med.uoa.gr (S.S.); apotiris@med.uoa.gr (A.P.); 3 Second Department of Obstetrics and Gynecology, Medical School, Aristotle University of Thessaloniki, 54640 Thessaloniki, Greece; cchatzakis@gmail.com (C.C.); e.vavoulidis@yahoo.com (E.V.); 4 Midwifery Department, Health Sciences School, International Hellenic University, 57400 Thessalonik, Greece; papasozomenou248@gmail.com; 5 First Department of Obstetrics and Gynecology, Medical School, National and Kapodistrian University of Athens, 11528 Athens, Greece; kdomali@yahoo.fr

**Keywords:** prenatal cannabis exposure, neurodevelopmental outcomes, maternal health risks, placental function, long-term developmental outcomes, public health policy

## Abstract

The use of cannabis during pregnancy has emerged as a mounting cause for concern due to its potential adverse consequences on both the mother and her offspring. This review will focus on the dangers associated with prenatal exposure to cannabis, particularly those related to neurodevelopment. It will also discuss the features of maternal and placental functioning that are likely to have long-term effects on the offspring’s development. The most pertinent and up-to-date materials can be found through a literature search. The literature emphasizes the substantial hazards associated with prenatal exposure to cannabis. These include impairments in cognitive function and difficulties in behavior in this particular instance. Structural and functional alterations in the brain can be noticed in offspring. The use of cannabis has been associated with an increased likelihood of experiencing pregnancy-related complications, such as giving birth prematurely and having a baby with a low birth weight. Additionally, it has been connected to potential negative effects on mental and emotional well-being. Studies have shown that when a pregnant woman is exposed to cannabis, it can negatively impact the functioning of the placenta and the growth of the fetus. This might potentially contribute to the development of placental insufficiency and restricted growth in the womb. Longitudinal studies reveal that children who were exposed to cannabis in the womb experience additional long-term developmental challenges, such as decreased cognitive abilities, reduced academic performance, and behavioral issues. In order to address the problem of cannabis usage during pregnancy, it is essential to adopt a comprehensive and coordinated strategy. This method should integrate and synchronize public health policy, education, and research initiatives. By implementing these targeted strategies, it is possible to mitigate potential health and welfare concerns for both present and future generations.

## 1. Introduction

Cannabis sativa-derived marijuana has garnered more attention the recent years because the plant is becoming legal for both medicinal and recreational use in different jurisdictions [1,2]. This converges with an increasing body of research that delves into the health implications that may relate to its use, especially for vulnerable populations like pregnant women. Taken together, the confluence of these factors places great emphasis on the need for a comprehensive examination of the risks associated with use during pregnancy.

It is well-known that pregnancy heralds a very special time with marked physiological and psychological change, during which maternal behaviors and exposures can yield lasting impacts on the health of the mother and fetus [3,4,5]. Against this background, the incidence of marijuana use by expectant mothers is hereby identified as a topic of great public health concern [6]. Epidemiological evidence seems to purport that the use of cannabis during gestational months is rather commonplace; it even ranges from 3% to 16% of its use worldwide and can reach higher levels among some demographic subgroups [6,7,8].

Prenatal exposure to cannabis has multi-generational implications and goes well beyond the immediate maternal and fetal health outcomes. Some evidence is emerging, however, to suggest that the use of cannabis during pregnancy may be linked to several adverse neurodevelopmental and obstetric complications, as well as long-term developmental outcomes in the offspring. The reason for understanding this, therefore, lays more emphasis on healthcare providers, policymakers, and researchers to realize the complex relationship between maternal cannabis and the health status of the perinatal period [9].

The following review will try to summarize the present literature on the risks of cannabis use in pregnancy, aiming to glean potential neurodevelopmental, obstetric, and long-term implications for prenatal exposure to cannabis by taking into consideration the results of epidemiologic, clinical, and preclinical studies. This review further seeks to bring out the importance of these findings to medicinal audiences in impressing upon them the informed clinical decision-making and evidence-based interventions that can be used to mitigate potential harms linked with cannabis use during pregnancy [10].

## 2. Materials and Methods

A literature search was performed using the MEDLINE and Scopus databases. The following terms were used in the search text fields (“Cannabis in Pregnancy” [Mesh]) AND “effects, risks, outcomes, factors, and health policies” [Mesh] AND (Cannabis*). The search algorithm was adjusted for each database while maintaining a common overall architecture. Relative published observational and interventional studies investigating cannabis use during pregnancy published up to March 2024 were included, while letters and commentaries were excluded.

**Figure 1:**
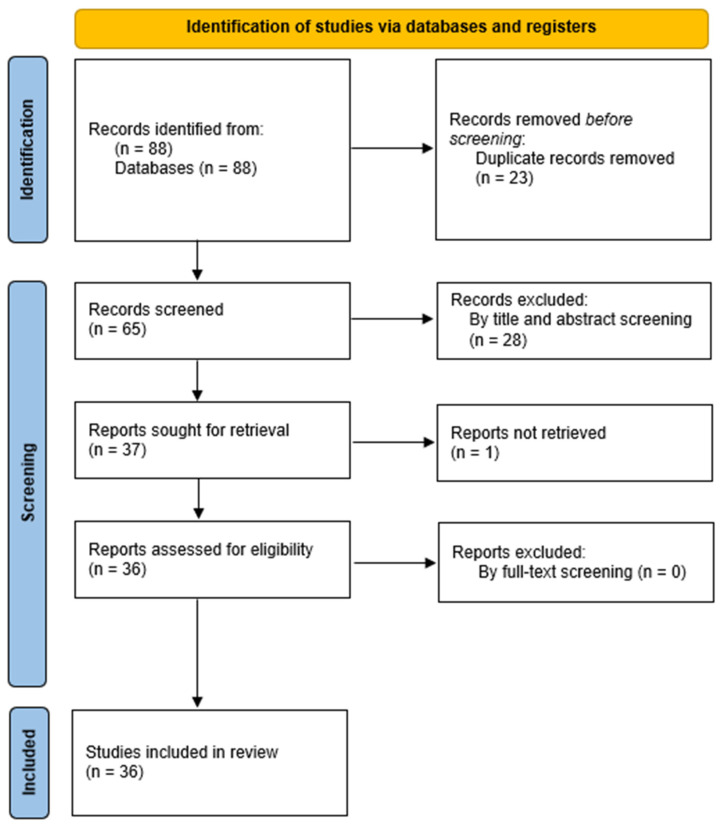
Flow diagram of the selection process of the included studies.

The review followed the PRISMA requirements (Preferred Reporting Items for Systematic Reviews and Meta-Analyses) but was not registered [11]. Retrieved records underwent semi-automatic deduplication by Rayyan [12]. Unique records were imported into Rayyan. Two independent authors screened them for relevance based on titles and abstracts only. Disagreements were resolved through consensus or by discussion with a third author. Articles deemed irrelevant were excluded, and the full-text copies of the remaining articles were assessed for eligibility as per the PICOS criteria by two blind reviewers. Inconsistencies were, once again, resolved by consensus or by a third reviewer. The references of the full-text copies were accessed to prevent the potential loss of eligible studies that were missed by the database search (snowball procedure). The process of selecting the studies is outlined in Figure 1. At first, 88 papers were found, and 65 were considered suitable for further screening after deleting duplicates. Afterward, these 65 articles were subjected to a title–abstract examination, and out of those, 37 articles satisfied the inclusion requirements. After a comprehensive evaluation of the full text, 36 papers met the criteria and were included in the current review.

## 3. Results

### 3.1. Cannabis and Neurodevelopmental Effects

One of the most focused and discussed issues of prenatal cannabis exposure is its neurocognitive effect on the developing fetal brain and later neuropsychological consequences among the offspring.

#### 3.1.1. Impact on Fetal Brain Development

Later studies reported that the effects of cannabis exposure on prenatal and fetal brain development are complex [13]. It is with the help of advanced neuroimaging techniques like magnetic resonance imaging (MRI) that many studies demonstrate different structural and connectivity brain differences in children exposed to cannabis in utero [14,15]. The study by El Marroun et al. reported a positive prenatal cannabis association with the thickening of the cortex within the frontal brain regions of young children. In contrast, prenatal tobacco was found to be related to the thinning of the cortex, primarily in the superior frontal and superior parietal cortices. These data suggest that exposure to both cannabis and tobacco during pregnancy can lead to changes in brain development through distinct harmful processes [14].

Further, Hurd et al. stated that there is a high tendency for strong interacting forces to be formed between THC and the cannabinoid receptors of the developing brain, possibly resulting in permanent alterations of the brain’s architecture and functions. Multiple studies have identified significant changes in brain regions associated with emotional regulation and cognitive function, such as the prefrontal cortex, in children who were exposed to cannabis during pregnancy.

According to Hurd et al. (2023), this will lead to an increased likelihood of experiencing anxiety, sadness, and behavioral problems in the future [15]. They elaborate that prenatal cannabis exposure can disrupt gene expression in brain development, which is crucial for the proliferation, differentiation, and branching of neurons. This can disrupt the normal progression of brain development, perhaps resulting in atypical cortical thickness and compromised white matter integrity. These structural changes will impact neural connections and functional brain networks that are responsible for cognitive and behavioral consequences.

#### 3.1.2. Cognitive and Behavioral Outcomes

Cognitive and behavioral studies have shed light on the effects of prenatal cannabis exposure. Prenatal exposure to cannabis has shown various epidemiological findings and is related to most cognitive deficits: deficits in executive functions, attention, and memory, among others. Such deficits are not transient; longitudinal cohort studies have shown that such deficits extend up to childhood, adolescence, and even advanced ages [16,17].

The effects of prenatal cannabis exposure are not only seen in the cognitive domain. Abnormalities in the behavior, such as impulsivity, hyperactivity, and emotional dysregulation, are also reported in the literature [14,18]. For instance, Hurd et al. demonstrated that genes responsible for the structure of cells in the brain and the production of important proteins that regulate behavior are negatively regulated by prenatal Δ9-tetrahydrocannabinol (THC) exposure [15]. This genetic effect is associated with an increase in the stress hormone cortisol, an increase in anxiety, heightened aggression, and disruptive behavior in exposed progeny. Furthermore, these behavioral changes are associated with changes in the structure of astrocytes, which are pivotal in facilitating brain function and communication.

Heavy use of marijuana during pregnancy has shown an association with negative perinatal outcomes, including growth restriction, preterm birth, and increased neonatal intensive care unit (NICU) admissions, as shown by Metz and Borgelt [19]. Although some studies showed no differences in the neonatal behavioral assessment scales, overall, the evidence leans toward significant long-term neurobehavioral consequences. Nomura et al. present compelling data on the compounded effects of prenatal cannabis exposure and environmental stressors [20]. More precisely, the authors used a sample of children who had been exposed to a substantial environmental stressor, Superstorm Sandy, and found that children exposed to maternal cannabis use and a substantial environmental stressor were at a 31-fold increased risk for disruptive behavioral disorders and a 7-fold increased risk for anxiety disorders. This is an interaction indicating increased risks in the case of several adverse exposures happening at the same time.

Such findings indicate that a complex, long-term relationship may exist between prenatal exposure to cannabis and neurobehavioral outcomes. Comprehensive longitudinal studies are needed to understand the mechanisms that underlie these effects and to devise effective prevention and intervention strategies. It is important to note that research on the cognitive effects of prenatal cannabis exposure remains mixed, with some studies reporting significant deficits in areas such as attention and executive function, while others, such as Brik et al. (2022), found no significant differences in cognitive abilities [21]. These discrepancies are explored further in Section 3.6.1.

#### 3.1.3. Mechanisms Underlying Neurodevelopmental Effects

Multiple studies suggest that the endocannabinoid system plays a role in the neurological effects of cannabis. These findings indicate that endocannabinoids and their receptors may play a role in regulating the neurobiological effects that occur during important stages of development [20,22].

Zou and Kumar went on to say that, in discussing the potential of cannabinoids in therapeutics, one should not forget that the endocannabinoid system also shows widespread expression and versatile functions [22]. They also described the importance of CB2 receptors and the interrelation between the endocannabinoid system and other neurotransmitter systems that may be able to modulate neurodevelopmental processes.

Additionally, Navarrete et al. indicated that nearly 3.8% of the global population had consumed cannabis, and it was well over 4.9% in pregnant women aged 15–44, spiking to 8.5% in the 18–25 age group, and this very high tendency towards cannabis use in pregnancy does create serious concerns regarding the impacts on fetal development [23]. Walker et al. have established the impact of psychoactive THC on the growth and development of the fetus [24], demonstrating that the exposure of the developing fetus to THC could restrict its growth by causing mitochondrial dysfunction and potentially disrupting trophoblast cell fusion. In mechanistic terms, THC treatment reduces the expression of markers that are associated with syncytialization and mitochondrial dynamics in human trophoblast cells without affecting cell viability.

Taken together, these findings suggest that exogenous cannabinoids disrupt the normal function of the endocannabinoid system during pregnancy and may exert detrimental effects on the cognitive and behavioral performances of offspring.

### 3.2. Cannabis and Risks of Maternal Health

The use of cannabis during pregnancy remains a health hazard for both the mother and the developing fetus.

#### 3.2.1. Pregnancy Complications

Studies suggest that marijuana use among mothers is significantly correlated to an increased likelihood of pregnancy complications that include preterm birth and low birth weight (LBW) [23]. A meta-analysis by Gunn et al. associated cannabis use by expectant women with a likely ratio of giving birth prematurely of 1.41 (95% CI 1.21–1.64) [25]. Gunn et al. indicated that among 24 reviewed studies, women who used cannabis in their pregnancy were at higher odds of having anemia (pooled OR= 1.36; 95% CI 1.10 to 1.69). They are also more likely to deliver babies with LBW, pOR = 1.77, 95% CI = 1.04 to 3.01, and to be admitted to NICU, pOR = 2.02, 95% CI = 1.27 to 3.21.

Similar findings were reported by Metz and Borgelt [19], who found maternal marijuana use in 2.7% of live births. Their study controlled by variables, including maternal age, race, and socioeconomic status (SES), showed that, although the composite adverse pregnancy outcomes were not significantly higher (31.2% vs. 21.2%; *p* = 0.14), there was still a significant cause for concern over the consequences of cannabis use. Finally, Brik et al. also reported vascular effects, with cannabis use predicting a higher UA pulsatility index, lower cerebral–placental ratio, and higher uterine pulsatility index at 33–35 weeks of gestation after correction for maternal factors [21]. These results suggest that screening for cannabis use during pregnancy is, thus, an important and potentially modifiable risk factor for adverse birth outcomes.

Cannabis use in pregnancy has been associated with several adverse obstetric outcomes, with the most consistent associations including an elevated risk for hypertensive disorders, placental abruption, and fetal growth restriction. Indeed, significant impairment of the placenta resulting from maternal cannabis use has been described, leading to reduced uterine blood flow and changes in fetal circulation that can result in compromised fetal growth and an increased risk of preterm birth [21]. In particular, Brik et al. (2022) demonstrated that intrauterine cannabis exposure is associated with significant alterations to maternal and fetal blood flow, as evidenced by increased uterine artery resistance and an altered fetal cerebral–placental ratio indicative of potential fetal hypoxia and growth restriction [21]. Also, hypertensive disorders, including the acquired form known as preeclampsia, are more frequent in pregnancies with cannabis exposure due to their vasoconstrictive effects on maternal vasculature. Apart from such vascular effects, impairment of normal placental function further increases the risk of placental abruption—a serious condition with the potential to compromise maternal and fetal health [13]. These findings also add to the evidence that health professionals should screen for the use of cannabis and counsel pregnant women appropriately on risks to maternal and fetal health.

#### 3.2.2. Impact on Maternal Mental Health

Another significant issue in the maternal use of cannabis is related to mental health, which forms a general factor about the mother. Recent studies have also suggested that prenatal exposure to cannabis was associated with higher mental health vulnerability. According to a longitudinal case study conducted by Metz et al., it was found that moms who used cannabis during pregnancy experienced increased depressive symptoms from the time they were pregnant until after giving birth [26]. The association persisted even after the possible confounding variables—maternal age, SES, and comorbid substance use—were controlled.

Further, maternal use of cannabis enhanced vulnerability to stress and anxiousness while pregnant. El Marroun et al. indicated that prenatal cannabis exposure enhanced susceptibility to anxiety disorders [27].

### 3.3. Cannabis and Its Effects on the Placenta

The placenta plays a major role as an exchange surface for the passage of nutrients and oxygen to the mother and the passage of waste from the developing fetus. A growing body of research is now revealing that the use of cannabis by mothers impairs placental function and results in poor subsequent fetal outcomes. The literature is hence current with an appraisal of the effects cannabis use has on placental physiology and fetal growth, focusing particularly on placental insufficiency in ItraUterine Growth Retardation (IUGR) development and its outcomes.

#### 3.3.1. Effects of Cannabis on Placental Function

It has been proven by numerous studies that cannabinoids can cross the placenta and directly affect its functionality and development. More specifically, it was demonstrated that THC exposure to placental tissues dramatically altered the levels of anandamide (AEA) and changed the gene expression profile of components of the endocannabinoid system, namely the AEA metabolic enzymes [28]. These changes in a duration-dependent manner of THC exposure disbalance the ratio between trophoblast apoptosis and hormone secretion, which is important for normal placental development and fetal growth. Importantly, these changes were generally observed at concentrations higher than those for recreational use.

Similarly, Walker et al. established that THC-induced placental adaptations limited fetal growth [24]. In a rat model, they could demonstrate symmetrical growth restriction in fetuses after in utero exposure to THC. They have reported an increase in mass in the labyrinth region of the placenta, indicating vascularization was disrupted. Indeed, Walker et al. have demonstrated that cannabis use during pregnancy is associated with narrower placental vascular networks, greater oxidative stress, and mitochondrial dysfunction. These changes are associated with SGA fetuses and could contribute to the long-term metabolic disorders observed in the offspring [24].

Costa et al. illustrated these mechanisms in more detail and indicated evidence to show that THC limits the turnover of the trophoblast by abrogating the needed processes in cytotrophoblasts for proliferation, differentiation, and apoptosis, which are necessary for placental development [29]. This failure of trophoblast remodeling and poor dynamics of placentation eventually leads to placental insufficiency, nutrients, and oxygen could not being well supplied to the developing fetus. Taken together, these reports provide empirical data that support their impact on placental and fetal development.

Table 1 reports some of the main findings, including placental weight, incidences of fetal growth restriction, and observations of placental morphology. Individual data points such as these are indicative of the physical impacts of prenatal cannabis exposure on major features of fetal development.

#### 3.3.2. Fetal Growth and Development Impact

Placental insufficiency, arising from maternal consumption of cannabis, has further been implicated in the pathogenesis of intrauterine growth retardation, characterized by poor growth and development of the fetus. Several epidemiologic investigations have reported IUGR in the aggregate. For instance, certain studies have shown that, even after adjusting for possible confounding factors, one is likely to arrive at an excess risk of delivering infants with LBW among mothers exposed to cannabis in utero [30].

Poor fetal outcomes other than LBW, such as small for gestational age (SGA) and preterm birth, have also been found to be associated with the effects of maternal cannabis use [30]. The findings of this meta-analysis, however, point to a dose-dependent relationship, meaning that with an increasing dose of cannabis exposure, the probability of preterm birth increases. Researchers also add that such findings may increase the potential adverse effects of cannabis on the growth and development of the fetus, hence implicating neonatal health and later outcomes.

#### 3.3.3. Role of Cannabinoids in Modulating Placental Physiology

The endocannabinoid system is a physiological process that is regulated through cannabinoid receptors (CB1 and CB2) and endogenous ligands in the form of endocannabinoids. The receptors are found in many tissues of both the mother and placenta. Studies have shown that the activation of the CB1 receptor by exogenous cannabinoids, such as THC, in the placenta leads to impaired endocannabinoid signaling. Subsequently, this results in aberrant functional placenta and growth restriction of the fetus. Indeed, emerging evidence has suggested that cannabinoids might interact with other signaling pathways implicated in placental physiology, including the mammalian target of the rapamycin (mTOR) pathway. According to various studies, the most recent study by the authors has strongly suggested that THC induces mTOR signaling in placental trophoblast cells and, therefore, takes a significant part in the regulation of protein synthesis, contributing to the effect of nutrients made by the placenta in their transport [28,29]. It is evident that the cannabinoid receptors crosstalk signaling pathways of the placenta concerning fetal development and intrauterine growth.

Generally, maternal cannabis use has been related to adverse effects on placental function and fetal growth, which could lead to placental insufficiency and intrauterine growth restriction with related fetal outcomes. More studies need to dissect the mechanisms of this regulation by cannabinoids and devise means that could bring relief from the negative effects of cannabis abuse in pregnancy.

### 3.4. Cannabis Dose–Response Relationships

An essential aspect of this knowledge would be dose–response associations of cannabis exposure during pregnancy with its potential health impacts on the mother and the fetus. Some other studies found to assess different levels of cannabis exposure have revealed results on its overall effect on neurodevelopment and placental function.

#### 3.4.1. Neurodevelopmental Effects

Dose–response effects have been examined between prenatal cannabis exposure and various neurodevelopmental outcomes in offspring (Table 1). For instance, the cohort study by Klebanoff et al. partitioned members into users whose mothers used high amounts of cannabis at least five times during pregnancy and others according to the quantity and number of times mothers used cannabis in pregnancy, studied the association between cannabis use in pregnancy and offspring neurodevelopmental outcomes [31]. The result showed a dose–response relationship, whereby the effect strength of the association between the use of cannabis and the neurodevelopment impairments risks increased with greater levels of exposure.

The dose-dependent effects on neurodevelopment have further been confirmed in animal studies. Based on rodent models, Costa et al. presented the effects of different concentrations of THC, the first psychoactive constituent of cannabis, on the synaptic plasticity and neurotransmitter activity of the developing brain [29]. The findings demonstrated alterations in dose-dependent neuronal connectivity and neurotransmitter systems that suggest possible mechanisms that would explain the observed neurobehavioral deficits associated with prenatal cannabis exposure.

#### 3.4.2. Placental Function and Fetal Growth

Some of the studies have studied dose–response relationships to cannabis exposure on placental function and fetal growth (Table 1). Prospective cohort studies on the relationship between maternal cannabis use and placental morphology, stratification for duration, and frequency of cannabis use by pregnant mothers were carried out [20,31,32]. The results revealed a dose–response effect: the higher the level of cannabis exposure, the greater the associations with the structural and functional differences in the placenta, such as vessel weight and vascularization.

They also added greatly in the survey of what effect differing concentrations of cannabinoids may have on fetal growth carried out a laboratory study on in vitro models to investigate the way THC influences the function of trophoblasts and the transport of nutrients in the placenta [33]. The findings have shown dose-dependent inhibition of trophoblast proliferation and nutrient transport, suggesting mechanisms by which cannabis exposure can compromise fetal growth and development.

The importance of the dose–response relationships in understanding the effects of cannabis exposure during pregnancy is an important area that may offer direction for clinical practice and public health policies. In this regard, there is evidence suggesting an increased risk for adverse maternal and fetal outcomes with high levels of cannabis exposure, such as neurodevelopmental impairments and the placenta (Table 1). This will require more inquiry into understanding the exact working mechanisms and developing interventions that can be more directed to lessen the probable deleterious effects of prenatal cannabis exposure.

### 3.5. Interaction with Other Substances

Precise estimation of cannabis use, including together with the use of other substances like tobacco or alcohol, will furnish a full understanding of its totality of effect on maternal and fetal health. However, reliance upon self-reported cannabis use does carry with it certain limitations in terms of accuracy. In fact, studies show that self-reporting generally understates actual cannabis consumption for either reasons of social desirability bias or concerns about legal and social repercussions [25]. Toxicology testing, in turn, may present a more objective method of detecting cannabis metabolites by showing patterns of use in a clearer manner. For instance, Costa et al., 2015, have demonstrated that toxicology testing may reveal higher and more frequent cannabis use than self-reports alone, especially in women who co-use tobacco or alcohol [34]. This difference is relevant for considering the interactive effects of cannabis with other substances since the risk of prenatal cannabis exposure may be increased by poly-substance exposure [34].

This is followed by the fact that the concurrent use of cannabis and tobacco among pregnant women is common, and their combined use is said to be associated with a distended risk of adverse obstetric outcomes. Similarly, the co-use of cannabis and alcohol has been repeatedly documented, and there is increased concern over compounded and adverse effects on fetal development [25,34]. Routine toxicology screening in prenatal screening can, therefore, enhance the vigilance of care providers for the recognition of at-risk pregnancies while individualizing interventions.

#### 3.5.1. Cannabis and Tobacco

This may be comorbidity with tobacco, which is also common during pregnancy. Even more worrying is a combination with other substances—there seems to be even greater worry among health workers because this may amplify negative outcomes. The use of both jeopardizes the health of the mother and the fetus on a high scale. Studies have noted that combining cannabis with tobacco greatly increased the risks of giving birth pre-term or having low-weight babies [35,36]. Additional investigations by Metz through systematic review further linked the habits to an increased likelihood of having placental abruption and stillbirth [19,26].

At this point it is hard for scientists to understand why these things mix so harmfully. Very likely, it is due to the more intricate ways the active compounds of cannabis and tobacco affect everything from placental function to fetal development. This will be the avenue for more work in the future that will help and guide pregnant women.

#### 3.5.2. Cannabis and Alcohol

The concomitant use of cannabis and alcohol is highly risky during gestation. Both substances can permeate the placental barrier and cause direct harm to the fetus. There is already a body of studies that have demonstrated such risks. The studies by El Marroun et al. showed that parents who concurrently used both cannabis and alcohol had a higher incidence of children with conduct problems [14,27].

The precise ways in which marijuana and alcohol co-exposure exacerbate these risks are not yet fully understood. It is suggested, however, that its combined use may increase the magnitude of their resultant adverse effects. According to Lees et al., through reviewing over 700 articles and 43 longitudinal studies, observed that both alcohol and cannabis heavy use was linked to small to moderate brain structure and function disturbances and neurocognitive impairment [37]. Alcohol heavy use led to a general decline in both volumes of gray matter and cortical thinning. On the other hand, heavy use of cannabis showed a reduced subcortical volume and an increase in frontoparietal cortical thickness. The combined use of the two substances usually yields a higher combined impact compared to the isolated use of just one substance.

Gunn et al. added that this interaction between cannabis and alcohol is a very complex issue [38]. Evidence points to substitution and complementary effects, considering patterns of use, timing, and characteristics of the user. It is in this complex environment that there is a need for more focused research into understanding how these potential drug interactions interact with maternal and fetal health. Women using cannabis in combination with other substances, such as tobacco and alcohol, during pregnancy also seem to be at the highest risk for adverse outcomes. This brought to the front the study by Carmichael et al., which underscored that the combined use of alcohol and cannabis is linked with unique predictors, consequences, and psychological processes than single substance use [39]. Hopefully, more of these interactions will be better understood as research continues, bringing forth more effective interventions and reducing the risks associated with poly-substance use during pregnancy to bring about safe outcomes for both mothers and children.

### 3.6. Long-Term Developmental Outcomes

Longitudinal studies make a great contribution to understanding the lasting effects of prenatal exposure to cannabis on offspring development. It tracks the cognitive function, academic attainment, and behavior trajectories right from infancy through childhood, adolescence, and into adult life. Interpretation of such findings from longitudinal studies, however, should humanly consider the methodological limitations and confounding variables intrinsic to such research efforts.

#### 3.6.1. Cognitive Function

One of the most disputed topics in the field of maternal–fetal health is that prenatal cannabis exposure has been associated with cognitive effects. However, this is an area of rather conflicting findings. As such, Brik et al. (2022) did not observe any significant difference in the cognitive faculties of children with and without prenatal cannabis exposure [21]. Similarly, a recent diverse birth–cohort study did not state any differences either in standardized test scores or in general academic performance, which could indicate that prenatal cannabis exposure does not universally impede cognitive function.

In contrast, some studies have found modest impairments in selected cognitive domains such as attention, executive functioning, and verbal comprehension. Odom et al., 2020 did find modest academic deficits among children who were exposed in utero to cannabis; there was noted to be a specific decrement in sustained attention and problem-solving capabilities [18]. Hooper et al. (2014) also reported that adolescents who had early and repeated prenatal exposure to cannabis showed significant deficits in attention and memory, with lower academic achievement, although their measured IQ did not appear to be affected [34]. Attention and executive function impairments are likely to impact cognitive activities such as school performance, which is discussed further in Section 3.6.2. Many of these inconsistencies in findings actually emanate from a number of aspects: differences in study design, measurement tools, and control for confounding variables.

For example, Brik et al. (2022) reported no significant cognitive differences with a relatively small sample size and limited follow-up [21], while Hooper et al. (2014), with longer follow-up into adolescence, showed attention and executive function deficits [34]. Differences in the measurement tools spur variability: Odom et al. (2020) focused on specific neuropsychological tests of attention [18], while Brik et al. (2022) used more general cognitive assessments [21].

In addition, SES, parental education, and environmental factors are well-known influences on cognitive results. In studies like Metz and Borgelt, 2018; Hurd et al., 2023, control over the aforementioned factors allowed a clearer interpretation of cannabis-exposure effects [15,19], while in some studies, such as that by Hooper et al. (2014) [34], there is incomplete accounting for them, possibly leading to biased results. Nomura et al. (2023) discussed poly-substance use, showing that the use of co-administration of alcohol and tobacco with cannabis had an adverse effect on cognitive deficits [20]. However, most studies have used smaller cohorts and shorter follow-up periods, including Brik et al. (2022) [21], that underestimated such effects.

All these environmental factors, genetic predispositions, and exposure to cannabis point to future studies using standardized methodologies that allow better control over the confounding variables to clearly depict the real cognitive effects of prenatal cannabis exposure.

#### 3.6.2. Academic Achievement

School-aged children who were exposed to cannabis in utero have been reported to perform poorly in school, usually showing evidence of significant learning challenges. Most of the longitudinal studies that relate the impact of prenatal cannabis exposure on children’s academic outcomes do not present a clear line of results. For example, the research by Schuetze et al. demonstrated no significant differences between groups regarding standardized testing or overall school performance [36]. Other data have even suggested a modest increase in academic difficulties, particularly in mathematics and reading comprehension [20].

Hooper et al. further complicated the picture by reporting that, compared with healthy peers, adolescents diagnosed with cannabis use disorder who were still using the drug showed significantly lower academic achievement [34]. Similarly, younger age of onset of regular cannabis use and higher maximum daily use were related to worse academic outcomes. Yet, the results were not wholly consistent, as the neurocognitive deficits seemed to improve or resolve under drug-free or prolonged abstinence situations, which left one with the possibility that the observed deficits might have had an origin in residual drug effects or another confounding factor.

Still, more comes from the study of polydrug use. For instance, Paramo et al. have looked at the combined effects of binge drinking and cannabis use on college academic achievement [40]. They demonstrated that co-using students, compared with their counterparts who either used just an elevated amount of alcohol or used neither of the substances, had a significantly worse GPA and poor academic adjustment. Notably, 34.33% of the variance in GPA accounted for difficulties in academic adjustment, meaning that combined substance use hinders successful adaptation in the academic environment.

Furthermore, Serrano et al. delved into the combined use of cannabis and tobacco on academic performance [41]. The students who used the two substances at the same time or concurrently had very low GPAs compared to the non-users. This trend was all over the categories of polydrug use, thus emphasizing the moderate yet stable relationship of combining cannabis and tobacco use on poor academic performance of university students.

#### 3.6.3. Behavioral Outcomes

Longitudinal studies disclose a considerable relationship between prenatal marijuana exposure and the development of behavioral problems in offspring, most of which occur in the guise of aggressive and delinquent conduct. To be more precise, a study by El Marroun et al. found that, by 18 months, the girls who were actively exposed to marijuana in their pre-natal stages showed increased aggressive behavior and attention problems [27]. The study found these associations robust to adjustments for potential confounders, including maternal age, socioeconomic status, and comorbid substance use.

A similar result was reported earlier by Schuetze et al. in 2019, who found prenatal exposure to cannabis related to behavioral problems to an extent, in that the level of these problems and their persistence varied [36]. This is a similar finding in that higher levels of externalizing behaviors have been reported in children exposed to cannabis prenatally compared to those not exposed. Still, these effects sometimes decrease when thought to be due to postnatal environmental effects such as the style of parenting and maternal mental health.

It is very complex how prenatal cannabis exposure interacts with conditions in the postnatal environment to give rise to such behavioral outcomes. The literature supports the interaction between prenatal exposures and postnatal conditions, including parental behavior, stress, and socio-economic status in general, to shape long-term behavioral development. In the words of El Marroun et al., “The mother’s cannabis use was associated with higher levels of stress and anxiety as well as with other adverse behavioral consequences in children”.

While there is evidence that prenatal cannabis exposure leads to cannabis-related behavioral problems in the offspring, the results are mixed and emphasized by this undercurrent of variability, which should encompass the host of influences that affect a part of the explanation. This should push for more research to understand the complex interplay of prenatal exposure on the postnatal environment in child development.

A separate study found a definite connection between exposure to cannabis during pregnancy and aggressive behavior and attention problems in girls. These behavioral issues are believed to be early signs of more serious mental health illnesses, such as depression and anxiety disorders, that may develop later in life. Their results showed that anxiety-related behaviors can be expected to be evident in children up to 18 months old when exposed to marijuana in the mother’s womb. It also underlies the fact that health professionals need to guide and provide antenatal education about the possible risks that might be incurred through cannabis use. Any such issue management with specified interventions would be highly supportive to gain less access to the harmful consequences related to prenatal exposure to cannabis. Additionally, public health policies will make populations aware of these risks and effectuate evidence-based interventions that promote and enhance maternal mental health [26].

### 3.7. Cannabis and Cultural and Socioeconomic Factors

Maternal use of cannabis during pregnancy is highly affected by a complex interplay with cultural and socioeconomic factors, which affect maternal behaviors and, most importantly, health outcomes. It underlines the knowledge and cultural competence of healthcare providers and the relevance of the policymakers’ developing interventions that can bridge these disparities.

#### 3.7.1. Cultural Influences

Cultural norms and beliefs differ drastically within different communities and populations; for example, substance use, such as cannabis, is acceptable, while in others, it is unwelcome. Other cultures may perceive cannabis as a traditional treatment that they have used to solve signs evident during pregnancy, like vomiting or pain, which they are not diagnosed properly, leading to its use irrespective of the perils that it may subject them to regarding maternal and fetal health [6,7]. Further, cultural acceptability regarding health-seeking of maternal healthcare services and access to prenatal services would influence whether the pregnant woman would report using cannabis [36].

Research has highlighted that the stigma is part of the culture with regard to substance use; therefore, when most pregnant women fear being judged or even receiving judicial judgment from the healthcare providers, they might not come out clearly about their use of cannabis [8]. Cultural beliefs could further fuel the attitude of women seeking help for substance use disorders while pregnant from problems associated with their role as women and motherhood.

#### 3.7.2. Socioeconomic Factors

SES contributes substantially to how cannabis use is patterned during pregnancy and may further magnify disparities in maternal and fetal health outcomes. Pregnant women of low SES undergo a greater number of stressors and have fewer resources, all together likely contributing to the raised rate of use of substances like cannabis [32,42]. This will further complicate and make worse economic instability, unemployment, and unavailability of affordable healthcare services that form these barriers to prenatal care and substance abuse treatment [36].

Such differences in education levels and healthcare access levels could potentially propagate pregnancy-related disparities in cannabis use. A pregnant woman with low education may have access to a lack of enough information on the risks of using cannabis or may be denied information and services to help her based on research evidence [43]. Some of these include structural barriers related to transport issues or language barriers, and they may lay obstacles to access to prenatal care and substance abuse treatment for populations from marginalized sectors [44,45].

### 3.8. Public Health Policy and Education

Public health policy and public education represent strongholds in the way toward the reduction of the risks related to cannabis use during pregnancy. Adopting evidence-based practices would see the public be aware and sensitized, the professionals in the health sector, and much harm averted towards better health for the mother and baby.

#### 3.8.1. Implications for Public Health Policy

The need for comprehensive public health policies focused on the prevention and reduction of cannabis use during pregnancy has increased. Policymakers should reconsider past policies that limit the access of pregnant women to cannabis products and provide cessation support programs. It is necessary to ensure that pregnant women and their health providers are properly armed with correct, evidence-based information regarding the risks of cannabis use during pregnancy.

Recent data also depict the rise in the potency of cannabis products as another factor of grave concern for maternal–fetal health. The potency of THC in cannabis products in the recent past has increased astronomically, going as high as 90% THC [46,47]. An increase in such potency has increased the cognitive and behavioral risks associated with prenatal cannabis exposure. Thus, it denotes an important call to action in terms of public health policy towards these trends.

Furthermore, the method of ingestion also potentially has an important impact on the potency and/or frequency of use. Studies have shown that vaporization is a prevalent method of consumption that permits higher concentrations of THC than smoking cannabis. Concentrates used with vaporizers usually reach over 80% THC, a far higher potential than smoked flower cannabis, which typically reaches a potency between 10% to 30% [48]. Therefore, the rapid onset and shorter duration of effects with inhalation routes of administration, such as smoking or vaping, can result in more frequent use and thus compound risks not only to the mother but also to the fetus. This suggests that policymakers must take into consideration not just the potency of cannabis products but also modes of ingestion when building public health interventions.

Possibly, reducing the allowable potency of cannabis products may serve as a vital and critical step for future public health intervention. Lessons learned from early legalization efforts, such as those in Colorado, have shown that increased THC potency is associated with increased risks, including mental health problems and cannabis-induced psychosis [46]. Placing restrictions on THC potency and improving labeling requirements as part of broader public health strategies may also help reduce risks to pregnant women and their offspring.

Moreover, policy strategies such as mandatory potency testing, warning labels, and advertisement restrictions have proven effective in the public dissemination of information and reduction of cannabis use [46]. If policymakers enacted such a requirement, coupled with regulations that restrict the potency of legal cannabis products, they would reduce the potential harmful effects linked to prenatal cannabis exposure.

#### 3.8.2. Strategies for Raising Awareness

Any public health effort directed at reducing cannabis use in pregnancy must necessarily involve education. The core of educating women fundamentally lies with healthcare providers, whereby they help pregnant patients understand the potential harms associated with the use of cannabis and support them in their cessation efforts. Educational programs specifically tailored to fill this gap are needed, as knowledge and training have still been shown to be insufficient among health professionals in several countries [8].

Actions like designing relevant materials or resources that will inform the expectant mothers and their health providers. These should be materials that clearly, appropriately, and accurately describe the risks of using cannabis in pregnancy and the best ways to provide cessation strategies for those considering changing their use, along with services that could offer support. This could also be shared in real-time using digital platforms and mobile applications that would link the expectant mothers to the right resources at the right time [49].

Education is of the essence in addressing the situation of prenatal cannabis use among both the provider and the pregnant woman, combined with potential policy changes. This will require targeted educational efforts that ensure the availability of accurate information on the potential risks associated with the use of cannabis and ready access to support services by the pregnant woman. Proper training is to be provided to healthcare providers for appropriate counseling of pregnant women in explaining the ill effects of cannabis and helping them to quit it.

#### 3.8.3. Advocating for Evidence-Based Approaches

Any of these interventions that are implemented as part of a public health initiative need to be evidence-based. Evidence-based interventions will stand as effective solutions to curb cannabis use and offer a benefit for better maternal and fetal health outcomes. The possible evidence-based strategies may involve the use of pharmacotherapy, psychosocial support services, and many different behavioral interventions [50].

Recent studies showed that motivational interviewing, cognitive behavioral therapy, and contingency management decrease the use of cannabis in this group of pregnant women [44,51]. The evidence created from such findings needs to be transferred into clinical practice and public health policy so that effective interventions are available to the greatest number of women possible. In summary, the risk of cannabis in the pregnant population will require the public health system to address it through policy and educational efforts (Table 1), aiming to decrease the harm while improving maternal and fetal health outcomes through evidence-based strategies.

**Table 1:** Summary of studies included in this review.

Section	Study	Study Details	Main Findings
3.1. Cannabis and Neurodevelopmental Effects			
Impact on Fetal Brain Development	[13]	SR/MA of human and animal studies assessing cannabis impacts on neurodevelopment, focusing on long-term behavioral and cognitive outcomes.	Prenatal CE can lead to altered brain development, including effects on ECS. This may result in cognitive deficits, impaired learning, memory issues, and increased risk for anxiety and depression.
	[14]	PCS of pregnant women examining prenatal CE and neurodevelopmental outcomes in children, with a focus on brain size and behavior.	Prenatal CE is associated with smaller HCB and increased risk of cognitive and behavioral problems during childhood, such as attention and memory deficits.
	[15]	MMS involving human observational data and animal models to assess long-term impacts on the dopaminergic system and behavior.	Prenatal CE affects the dopaminergic system, which may lead to increased impulsivity, altered reward processing, and a higher risk of substance use disorders in later life.
Cognitive and Behavioral Outcomes	[18]	CSS on CU during pregnancy and its association with neurodevelopmental outcomes in offspring, including specific cognitive and behavioral assessments.	Prenatal CE correlates with higher rates of attention disorders and cognitive deficits, with specific risks depending on the timing and frequency of CU.
	[19]	SR/MA of existing CSs focusing on over 4000 women exposed to cannabis during pregnancy.	CU during pregnancy is linked to preterm birth, lower GBW, and adverse neurodevelopmental outcomes.
	[20]	Longitudinal CS tracking maternal CU and prenatal stress exposure (Superstorm Sandy), with diagnostic interviews for child psychopathology.	Children exposed to both prenatal CU and prenatal stress were at increased risk of developmental psychopathology, with 31-fold higher odds of disruptive behavior disorders and a 7-fold increase in anxiety disorders.
Mechanisms Underlying Neurodevelopmental Effects	[22]	SR/MA of CTs and OSs involving pregnant women who used cannabis and their offspring.	The review highlights the ECS’s role in neurodevelopment and suggests that prenatal CE may impair neurodevelopment, particularly in cognition and behavior, through alterations in the ECS.
	[23]	Comprehensive SR/MA of clinical and animal data on behavioral and neurobiological effects of CE in pregnant women, focusing on long-term impacts on offspring’s neurodevelopment.	Prenatal and postnatal CE are linked to neurobiological disruptions, including disturbances in attention, memory, and emotional regulation, as well as increased aggressiveness.
	[24]	Longitudinal CS with 12,000 children (age 9–10) being tracked through neuroimaging studies and assessments of cognitive and behavioral outcomes.	Prenatal CE is associated with cognitive and behavioral challenges in adolescence, particularly related to executive functions and attention problems.
3.2. Cannabis and Risks of Maternal Health			
Impact on Maternal Mental Health	[26]	Prospective OS assessing pregnant women using cannabis (n = 384). Data were collected using self-reports and clinical measures, with obstetric outcomes and placental assessments conducted via Doppler ultrasound.	Maternal CU during pregnancy was associated with an increased risk of preterm birth, GBW, and NICU admissions. There were also reports of increased vascular resistance in the placental bed, affecting fetal blood flow.
	[27]	PCS with 974 mothers utilizing questionnaires and clinical evaluations to assess CE and child outcomes.	CU during pregnancy was associated with behavioral problems in children at ages 18 months and 3 years. The findings suggested that prenatal CE can influence early childhood behavior, particularly emotional reactivity.
3.3. Cannabis and Its Effects on the Placenta			
Effects of Cannabis on Placental Function	[28]	A CSS of pregnant women using cannabis (n = 251) utilizing Doppler measurements of umbilical and uterine arteries to assess blood flow resistance in pregnancies exposed to cannabis.	CE led to increased vascular resistance in the umbilical artery, fetal growth restriction, and low GBW.
	[29]	An RS of 137 pregnant women, split between cannabis users and non-users, assessing birth outcomes and placental function through clinical measures and self-reported cannabis use.	CU was linked to poor placental function, higher rates of preterm birth, and NICU admissions.
Fetal Growth and Development Impact	[30]	Large-scale OS of 1200 pregnant women using survey data and clinical records to assess cannabis use patterns and birth outcomes.	Prenatal CU was associated with increased odds of neonatal complications, including preterm birth and low GBW. Co-use of alcohol and cannabis significantly exacerbated these risks.
3.4. Cannabis Dose-Response Relationships			
Neurodevelopmental Effects	[31]	RS using self-reported CU and clinical birth outcome data from 1562 pregnant women, adjusted for socioeconomic factors.	CU during pregnancy was significantly associated with preterm birth and low GBW. Women who used cannabis were also more likely to develop gestational hypertension.
	[52]	Ex vivo dual placental perfusion, in vivo Doppler ultrasound assessments of umbilical artery flow to assess placental vascular resistance and vasodilatory responses.	In pregnancies with FGR, placentas exhibited higher vascular resistance and reduced flow-mediated vasodilatation, which correlated with poor fetal growth outcomes.
	[53]	CSS of pregnant women in China (n = 5000+) focused on cannabis and tobacco use during pregnancy.	CU was associated with a significant increase in adverse birth outcomes, including preterm birth and low GBW.
	[54]	Longitudinal CS (n = 3500 pregnant women) with both self-reported data and sample verification.	Significant associations were found between CU and opioid use during pregnancy, with adverse effects on fetal neurodevelopment.
	[32]	PCS of 1200 pregnant women with self-reported CU and child development assessments post-birth.	Early CE was linked to higher risks of mental health disorders in offspring, particularly anxiety and depression.
3.5. Interaction with Other Substances			
Cannabis and Tobacco	[25]	SR/MA synthesizing data from 24 CS and case–control studies.	Strong evidence linking prenatal CE to preterm birth and low GBW, as well as increased NICU admissions.
	[34]	Longitudinal study with neuropsychological assessments of children followed for up to five years post-birth.	Prenatal CU is associated with delayed cognitive and behavioral development in offspring.
	[35]	Population-based RCS study using registry data involving over 10,000 women.	Significant increase in CU among pregnant women over a 5-year period, with the highest rates among young, low-SES women.
	[36]	Qualitative and quantitative data collection from 1500 families with a history of CU and child development assessments	Maternal CU negatively impacted family dynamics and child developmental outcomes.
Cannabis and Alcohol	[37]	SR/MA of longitudinal studies and CSSs examining the neurodevelopmental impact of CU.	CU during adolescence is associated with neurodevelopmental changes, including alterations in brain structure and function, particularly in the prefrontal cortex.
	[39]	Survey and self-report data were analyzed using regression models to assess the link between context and CU outcomes.	The frequency and context of CU can predict behavioral outcomes, including substance abuse and mental health issues.
3.6. Long-Term Developmental Outcomes			
Academic Achievement	[41]	CSS using questionnaires to measure stress, anxiety, and CU, both cannabis users and non-users.	CU is associated with higher levels of stress and anxiety among college students.
	[40]	Longitudinal CS assessing cognitive functions using neuropsychological tests.	Early CU is linked to cognitive deficits, including attention and memory issues, which persist into adulthood.
Behavioral Outcomes	[45]	PS using developmental scales to assess infant behavior and health compared to non-exposed infants.	Prenatal CE is associated with poorer developmental outcomes in infants, such as lower GBW and higher irritability.
3.7. Cannabis and Cultural and Socioeconomic Factors			
Cultural Influences	[7]	RCS study examining birth outcomes in cannabis-using mothers compared to non-users.	CU during pregnancy is linked to an increased risk of preterm birth and NICU admissions.
	[8]	CSS of self-reported cannabis and tobacco use among pregnant women.	Co-use of cannabis and tobacco during pregnancy increases the risk of adverse birth outcomes, such as low GBW and preterm delivery.
Socioeconomic Factors	[42]	CS with neurodevelopmental assessments, including cognitive and behavioral evaluations.	Prenatal CE can lead to subtle cognitive and behavioral issues, though results are inconsistent.
	[44]	SR/MA of 26 studies, analyzed depression scales and substance use.	Substance use during pregnancy is associated with an increased risk of PDD (OR = 3.67)
3.8. Public Health Policy and Education			
Implications for Public Health Policy	[55]	Analyzed National Survey on Drug Use and Health data using logistic regression (n = 8713 pregnant women).	Increased marijuana use among pregnant women by 62% over the decade, affecting risk perception.
	[56]	Comprehensive SR/MA and synthesis of studies focusing on substance abuse and neurodevelopmental outcomes.	Substance use disorders and mental health linked to impaired cognitive and emotional development in children.
Strategies for Raising Awareness	[49]	Logistic regression of trends in risk perception based on survey data.	19% of pregnant women reported no perceived risk of regular marijuana use, increased from 4.6% in 2005.
	[50]	SR/MA focused on substance use and PPD risk.	Substance use during pregnancy increases the risk of PPD by 29%.

CE: cannabis exposure; CS: cohort study; CSS: cross-sectional study; CT: clinical trial; CU: cannabis Use; ECS: endocannabinoid system; FGR: fetal growth restriction; GBW: gestational birth weight; HCB: head circumference at birth; MMS: multi-modal study; OS: observational study; NICU: neonatal intensive care unit; PCS: prospective cohort study; PPD: postpartum depression; RS: retrospective study; SES: socioeconomic society; SR/MA: systematic review and meta-analysis.

## 4. Discussion

This review clearly indicates that prenatal exposure to cannabis has long-term consequences that extend beyond the immediate effects on the health of both the mother and the fetus. There is growing evidence indicating that using cannabis while pregnant may be associated with various negative effects on the development of the brain and nervous system, as well as problems during pregnancy and long-term developmental issues in the child. This review aims to provide a comprehensive summary of the existing literature on the risks associated with cannabis use during pregnancy by presenting the potential impact on neurodevelopment, obstetric outcomes, and long-term consequences of prenatal exposure to cannabis.

Cannabis exposure during pregnancy has been linked to neurocognitive impacts on the developing fetal brain and subsequent neuropsychological outcomes in the offspring. Exposure to both cannabis and tobacco during pregnancy can result in alterations in brain development through separate detrimental mechanisms, leading to cognitive impairments, executive functioning, attention, memory, and irregularities in behavior. The endocannabinoid system is involved in the neurological effects of cannabis, and external cannabinoids can interfere with the normal functioning of the endocannabinoid system during pregnancy, potentially leading to negative consequences on the cognitive and behavioral abilities of the offspring.

Maternal cannabis consumption hampers the functioning of the placenta and leads to unfavorable effects for the fetus. Research indicates that cannabinoids have the ability to traverse the placenta and directly impact its functionality and development. The concurrent use of cannabis, nicotine, and alcohol during pregnancy is frequently associated with an elevated likelihood of preterm birth, low birth weight, placental abruption, and stillbirth.

Longitudinal research has yielded useful insights into the enduring impact of prenatal cannabis exposure on the development of offspring, but the conclusions must take into account methodological constraints and factors that may influence the results. Cultural and socioeconomic elements also play a role in the utilization of cannabis during pregnancy, with cultural acceptability and stigma influencing the willingness of pregnant women to reveal cannabis usage. Effective implementation of public health policy and comprehensive education programs are essential in mitigating the potential hazards associated with cannabis consumption during pregnancy.

This study tries to emphasize the significance of these findings for medical professionals, highlighting the relevance of educated clinical decision-making and evidence-based treatments to reduce potential risks associated with cannabis use during pregnancy.

It is important for healthcare practitioners, policymakers, and academics to grasp the intricate connection between maternal cannabis use and the health of the perinatal period.

## 5. Conclusions

This review demonstrates that the use of cannabis has been associated with an increased likelihood of experiencing pregnancy-related complications, such as giving birth prematurely and having a baby with a low birth weight. It is also connected to potential mental health consequences. Studies indicate that exposure to cannabis during pregnancy can impact the functioning of the placenta and the growth of the fetus. This could potentially contribute to the development of placental insufficiency and restricted intrauterine growth. Longitudinal studies exacerbate the long-term developmental challenges faced by children who were exposed to cannabis while in the womb, such as decreased cognitive abilities, diminished academic performance, and behavioral issues.

The problem of cannabis use in pregnancy can only be adequately addressed through a comprehensive and coordinated approach that involves and aligns public health policy, education, and research efforts. These targeted strategies may help avert the future health and well-being of young and the next-born generations.

## Data Availability

This study did not create or analyze new data, and data sharing does not apply to this article.
